# Declarative and Non-declarative Memory Consolidation in Children with Sleep Disorder

**DOI:** 10.3389/fnhum.2015.00709

**Published:** 2016-01-11

**Authors:** Eszter Csábi, Pálma Benedek, Karolina Janacsek, Zsófia Zavecz, Gábor Katona, Dezso Nemeth

**Affiliations:** ^1^Institute of Psychology, University of SzegedSzeged, Hungary; ^2^Heim Pal Children’s HospitalBudapest, Hungary; ^3^MTA-ELTE NAP B Brain, Memory and Language Research Group, Institute of Cognitive Neuroscience and Psychology, Research Centre for Natural Sciences, Hungarian Academy of SciencesBudapest, Hungary; ^4^Institute of Psychology, Eotvos Lorand UniversityBudapest, Hungary

**Keywords:** sleep deprivation, memory consolidation, declarative memory, skill learning, sequence learning, sleep-disordered breathing (SDB), implicit learning

## Abstract

Healthy sleep is essential in children’s cognitive, behavioral, and emotional development. However, remarkably little is known about the influence of sleep disorders on different memory processes in childhood. Such data could give us a deeper insight into the effect of sleep on the developing brain and memory functions and how the relationship between sleep and memory changes from childhood to adulthood. In the present study we examined the effect of sleep disorder on declarative and non-declarative memory consolidation by testing children with sleep-disordered breathing (SDB) which is characterized by disrupted sleep structure. We used a story recall task to measure declarative memory and Alternating Serial Reaction time (ASRT) task to assess non-declarative memory. This task enables us to measure two aspects of non-declarative memory, namely general motor skill learning and sequence-specific learning. There were two sessions: a learning phase and a testing phase, separated by a 12 h offline period with sleep. Our data showed that children with SDB exhibited a generally lower declarative memory performance both in the learning and testing phase; however, both the SDB and control groups exhibited retention of the previously recalled items after the offline period. Here we showed intact non-declarative consolidation in SDB group in both sequence-specific and general motor skill. These findings suggest that sleep disorders in childhood have a differential effect on different memory processes (online vs. offline) and give us insight into how sleep disturbances affects developing brain.

## Introduction

Healthy sleep is critical for children’s cognitive, behavioral, and emotional development. Unfortunately, sleep disturbances are common in childhood, including both primary (e.g., insomnia and sleep apnea) and secondary sleep disorders (other illnesses e.g., depression or bad/altered sleep hygiene results the sleep disorders; Anuntaseree et al., 2001; Rosen et al., 2003; Bixler et al., 2009). The prevalence of sleep disorders in childhood estimates vary from 0.7 to 13% (Brunetti et al., 2001; Bixler et al., 2009). Therefore clinical research and practice need to focus more on sleep disturbances in children. The current study focuses on the effect of childhood sleep-disordered breathing (SDB) on declarative and non-declarative memory consolidation.

Memory consolidation can be defined as a set of processes whereby the newly acquired and initially labile memory traces become more stable with the passage of time (Stickgold and Walker, 2007; Spencer, 2013; Urbain et al., 2013). Growing body of evidence indicates that sleep plays a crucial role in these consolidation mechanisms and leads to memory representation being more resistant to interference and forgetting (Dorfberger et al., 2007; Diekelmann et al., 2009; Rudoy et al., 2009; Diekelmann and Born, 2010; Diekelmann, 2014; Mednick et al., 2011; Born and Wilhelm, 2012; Schönauer et al., 2014, 2015).

The effect of sleep on declarative (e.g., remembering events or facts) and non-declarative/procedural memory (e.g., learning languages, learning to musical instruments and movement-based sports) domains is well explored in healthy adults (Fischer et al., 2002; Walker et al., 2002; Gais and Born, 2004; Gais et al., 2006; Song et al., 2007; Rickard et al., 2008; Nemeth et al., 2010), but only a few studies focused on children. These studies with typically developing children found that post-training sleep facilitates the consolidation of declarative memory processes (Gais et al., 2006; Backhaus et al., 2008; Wilhelm et al., 2008; Prehn-Kristensen et al., 2009) but the effect of sleep on non-declarative memory consolidation is still controversial. Some studies failed to find a facilitating effect of sleep on non-declarative memory consolidation (Wilhelm et al., 2008; Prehn-Kristensen et al., 2009), however some recent studies revealed that sleep impacts on non-declarative/procedural memory in children (Fischer et al., 2007; Wilhelm et al., 2012, 2013; Urbain et al., 2013). In contrast to these results, Fischer et al. (2007) demonstrated offline decrement after sleep in non-declarative memory in children compared to adults who showed offline improvement after sleep. In a recent study Borragán et al. (2015) clarified the picture by showing that sleep has a beneficial effect on the consolidation of motor skills but it has no influence on sequential skills. These results indicate that sleep-dependent non-declarative memory consolidation can depend on age (Fischer et al., 2007; Wilhelm et al., 2008) and the nature of the task (Wilhelm et al., 2008, 2012; Borragán et al., 2015). Less is known about how permanent sleep-disorder influences sleep-dependent consolidation of declarative and non-declarative memories in children.

In our study we examined children with SDB which is an ideal population to investigate the effects of sleep disorder on the consolidation of different memory systems. SDB is a spectrum disorder characterized by prolonged and intermittent partial (such as snoring) or complete upper airway obstruction (such as Obstructive sleep apnea, OSA) that disturbs normal ventilation and sleep pattern during sleep. Especially slow wave sleep and REM sleep are affected in SDB (Coleman, 2003; Li and Lee, 2009; Sinha and Guilleminault, 2010). OSA is the worst grade on this spectrum characterized by repetitive episodes of complete or partial upper airway obstruction during sleep resulting hypoxia and fragmented sleep (Banno and Kryger, 2007). The main cases of SDB in children is with adenotonsillar hypertrophy, but it also occurs with obesity, upper airway narrowing due to craniofacial or neuromuscular abnormalities or muscular coordination (Arens et al., 2001; Guimaraes et al., 2008; Katz and D’Ambrosio, 2008).

The neurocognitive consequences of SDB in children have not yet been fully evaluated. There is emerging evidence that cognitive deficits are most consistently apparent on tasks involving sustained attention and executive functions (Beebe and Gozal, 2002; Archbold et al., 2004; O’Brien et al., 2004b; Beebe, 2006). In addition, SDB is associated with deterioration of memory; for example, Gottlieb et al. (2004) revealed that children with SDB had significantly poorer performance on verbal (Narrative Memory) and visual memory tasks (Memory for Faces) compared to healthy participants. Kheirandish-Gozal et al. (2010) investigated the learning before sleep (acquisition) and delayed free recall performance after an overnight sleep (retention) in children with OSA compared with children without sleep disorder. They used pictorial-based memory task where the subjects required to learn and remember animal pictures. They found that both immediate (before sleep) and delayed recall performances (after sleep) were worse among OSA children compared to the control subjects. The authors suggested that this reduced performance may be caused by impaired ability to use adequate learning strategies which either leads to difficulties to learn new information or children with OSA suffer from impaired encoding or altered retrieval. In our recent study (Csábi et al., 2013), we investigated declarative and non-declarative memory performance in one learning session (without consolidation) and showed weaker declarative but intact non-declarative memory performance in children with SDB compared to the controls. These results suggest that the more attention-demanding declarative learning are more vulnerable to permanent sleep disorder than less attention demanding non-declarative learning.

The mechanisms causing these neuropsychological deficits have not been fully delineated. Previous studies suggest that the developing central nervous system in children may be relatively more vulnerable to the fragmented sleep and hypoxia, particularly the hippocampus and frontal lobe structures (Macey et al., 2002; Morrell et al., 2003; Bartlett et al., 2004; Halbower et al., 2006; Owens, 2009). Children with SDB can exhibit daytime behavioral regulation problems (such as inattention, hyperactivity, aggressiveness, social withdrawal) which might also imply frontal lobe dysfunction (Chervin and Archbold, 2001; Beebe and Gozal, 2002; Archbold et al., 2004; Archbold, 2006).

Previous studies examined memory encoding and consolidation before and after sleep in patients with sleep apnea in adults, and showed that declarative and some aspects of non-declarative memory performance is affected in patients with OSA (Kloepfer et al., 2009; Djonlagic et al., 2012; Csabi et al., 2014). Similarly to Borragán et al. (2015) we found dissociation in the effect of sleep (and/or sleep disorder) on offline changes of general motor skills and sequence-specific learning: adult OSA patients showed impaired consolidation of general motor but not on sequence-specific learning (Csabi et al., 2014). To our knowledge, the current study is the first to assess the effects of sleep disorder on declarative and non-declarative memory functions before and after a nighttime sleep in children. Based on previous studies, we hypothesized that SDB in childhood has an adverse effect on the consolidation of declarative memory while it has less influence on non-declarative memory consolidation. Within the later one we expect differences in the consolidation of motor and sequence-specific aspects of the offline changes.

## Materials and Methods

### Participants

Thirty two children participated in the experiment. Breathing events during sleep, Body Mass Index (BMI) and working memory (WM) measures of the SDB patients and healthy participants are listed in Table 1. All participants underwent an overnight polygraphy, which was performed with the Somnomedics Somnoscreen plus device (Randersacker, Germany) at the Sleep Disorders Laboratory of Heim Pál Children’s Hospital, Budapest, Hungary. Patients who met the International Classification of Sleep Disorders criteria’s (American Academy of Sleep Medicine, 2001) for SDB were included in the study. SDB was diagnosed by a board-certified sleep physician. The SDB group consisted of sixteen children with SDB (average age: 8.56 years [min: 6 to max: 11 years], *SD*: 2.31; 6 females/10 males) six of them with OSA and ten of them with primary snoring. The Apnea/Hypopnea (AHI) index of the OSA patients (*M* = 17.32, *SD* = 30.54, range 2–79) was significantly higher (all *p*’s < 0.01) than that of the snoring patients (*M* = 0.11, *SD* = 0.19, range 0–1) as well as the controls (*M* = 0.11, *SD* = 0.20, range 0–1). Similarly, the snore index of the snoring patients (*M* = 55.10, *SD* = 54.95, range 6–155) was significantly higher (all *p*’s < 0.03) than that of the OSA patients (*M* = 16.67, *SD* = 28.52, range 0–73) as well as the controls (*M* = 0.13, *SD* = 0.34, range 0–1). According to the literature, the neurobehavioral deficits is associated with snoring in children are similar to those found in children with OSA (Gozal and O’Brien, 2004; O’Brien et al., 2004a). Therefore we compared the performance of the SDB group to that of controls and did not intend to examine the OSA and snoring subgroups separately. All SDB patients were untreated prior to and during the experimental night in the sleep laboratory.

**Table 1:** **Means (standard deviations) of participants’ data are presented in the table**.

	Control (*n* = 16)	SDB (*n* = 16)	*t* (df)	*p*-value
Snore index events/hour	0.13 (0.34)	40.69 (49.52)	−3.28 (15.001)	0.005**
AHI event/hour	0.11 (0.20)	6.56 (19.62)	−1.31 (15.003)	0.21
Max. desaturation (%)	92.31 (4.13)	90.56 (7.75)	0.80 (30)	0.43
Desaturation index (%)	0.56 (0.89)	11.25 (26.76)	−1.60 (15.003)	0.13
BMI kg/m^2^	15.19 (1.22)	19.25 (5.17)	−3.06 (16.67)	0.01*
Counting span	2.88 (0.72)	2.48 (0.55)	1.74 (30)	**0.09^+^**
Listening span	2.40 (0.75)	2.16 (1.09)	0.72 (30)	0.48
Digit span	4.81 (0.65)	4.50 (0.89)	1.13 (30)	0.27

Snore Index: snoring events per hour; AHI: Apnea-Hypopnea Index: apnetic and hypopnetic events per hour of sleep; Max. desaturation: ratio of oxihemoglobin to the total concentration of hemoglobin present in the blood; Desaturation Index: number of time/hour of sleep that the blood’s oxygen level drops by 3% or more for baseline; BMI: body mass index kg/m^2^. Listening Span Task: a working memory (WM) task in which the participants are required to listen to increasingly longer sequences of sentences and to recall the final word of all the sentences in each sequence in serial order (Daneman and Blennerhassett, 1984). Counting Span Task: a WM task in which participants are required to count a growing number of colored dots on the computer screen and remember the number of the dots of each sequence (Case et al., 1982; *p < 0.05, **p < 0.01, ^+^p < 0.10).

The control group consisted of sixteen healthy participants (average age: 8.75 years, *SD*: 1.44 [min: 6 to max: 15 years]; 8 females/8 males). The control and the patient groups were matched on age (*t*_(30)_ = 0.28, *p* = 0.78) and gender ($\chi_{\left(1\right)}^{2}$ = 0.51, *p* = 0.48) and parental education (mother education: *t*_(12.54)_ < 0.001, *p* > 0.99; father education *t*_(23)_ = 0.61, *p* = 0.55). They did not suffer from any developmental, psychiatric or neurological disorders, and were free of any sleeping disorders. Informed written parental consent and verbal assent of the children were provided, and participants did not receive any financial compensation for their participation. Ethics approval was obtained by the Ethics Committee at Heim Pal Children’s Hospital, Budapest.

## Tasks

### Tasks

#### Story Recall-“The War of the Ghosts” Test

Declarative memory performance was measured by “The War of the Ghosts” test (Bartlett, 1932; Bergman and Roediger, 1999). This is a story recall test, which is widely used to measure declarative memory for episodes (Bartlett, 1932; Bergman and Roediger, 1999; Andreano and Cahill, 2006, 2008; Schwabe and Wolf, 2009; Hardt et al., 2010). In this test children are asked to listen and repeat the story after various intervals (immediately or after a determinate interval). The story consisted of 36 sentences; based on the standardized scoring, each sentence is allocated 1 point for the verbatim recalled sentences and 0.5 points for partly correct responses (gist of the sentences; Bartlett, 1932; Gauld and Stephenson, 1967; Csábi et al., 2013).

#### Alternating Serial Reaction time (ASRT) Task

We used a modified version of the original ASRT task in order to assess non-declarative/procedural learning performance. In the original version of this task, four open circles were displayed in the middle of the computer screen and subjects had to press the corresponding button when the circles were filled in with black (Howard and Howard, 1997). In our version, a dog’s head appeared in one of the four empty circles on the screen and participants had to press the corresponding button (Nemeth et al., 2010). The computer was equipped with a special keyboard with four marked keys (Y, C, B and M on a QWERTZ keyboard; thus, compared to the English keyboard layout, the location of the buttons Z and Y were switched), each corresponding to one of the horizontally aligned circles. Before beginning the task, detailed instructions were read to the participants. We emphasized that the aim was to try to respond as quickly and as correctly as possible. Session 1 (Learning Phase) consisted of 25 blocks, with 85 key presses in each block—the first five stimuli were random for practice purposes, then an eight-element alternating sequence (e.g., 2r1r4r3r, where numbers represent the four places on the screen, and r represents an event randomly selected from the four possible places) repeated ten times. This sequence structure is often described as non-adjacent second-order dependency (Remillard, 2008). Similarly to earlier studies (Nemeth et al., 2010), stimuli were presented 120 ms after the previous response (response-to-stimulus interval, RSI). Each block required about 1.5 min and the entire session took approximately 30–40 min. Between blocks, participants received feedback about their overall RT and accuracy on the screen and then rested 10–20 s before starting a new block. Session 2 (Testing Phase) consisted of 5 blocks; the number of key presses and the RSI were the same as in Session 1 and this Testing Phase took approximately 5–10 min to complete.

A different ASRT sequence was selected for each participant based on a permutation rule so that each of the six unique permutations of the four repeating events occurred. Consequently, six different sequences were used across participants.

As there is a fixed sequence in the ASRT task alternating with random stimuli (for instance 2r1r4r3r), some triplets or runs of three stimuli occur more frequently than others. For example, in the above illustration, triplets 2_1, 1_4, 4_3, and 3_2 would occur often because the third element could be derived from the sequence or could also be a random element. In contrast, 1_2 or 4_1 would occur less frequently because in this case the third element could only be random. Following previous studies (Howard and Howard, 1997; Song et al., 2007; Nemeth et al., 2010), we refer to the former as high-frequency triplets and the latter as low-frequency triplets. Out of the 64 possible triplets, the 16 high-frequency triplets occurred 62.5% of the time and the 48 low-frequency triplets occurred 37.5% of the time. Note that the final event of high-frequency triplets is therefore more predictable from the initial event compared to the low-frequency triplets.

Previous studies have shown that as people practice the ASRT task, they come to respond more quickly to the high- than low-frequency triplets, revealing sequence-specific learning (Howard and Howard, 1997; Howard et al., 2004; Song et al., 2007; Nemeth et al., 2010; Janacsek et al., 2012). In addition, general motor skill learning is revealed in the ASRT task by the overall speed-up due to practice, irrespective of the triplet types. Thus, using the ASRT task enables to measure both sequence-specific and general motor skill learning.

#### Procedure

There were two sessions in the experiment. The declarative and non-declarative performance was assessed at 7–9 PM prior to sleep (Learning Phase/Session 1) and 7–9 AM after sleep (Testing Phase/Session 2), thus the average interval between the Learning and Testing Phase was 12 h. The order of the administration of declarative and non-declarative tasks was counterbalanced in order to minimize the interference between declarative and non-declarative tasks (see Brown and Robertson, 2007).

#### Statistical Analysis

To facilitate data processing, the blocks of ASRT were organized into epochs of five blocks. The first epoch contained blocks 1–5, the second epoch contained blocks 6–10, etc. We calculated mean accuracy and median RT for correct responses only; separate for high- and low-frequency triplets and for each subject and each epoch. Note that for each response (n), we defined whether it was a high- or a low-frequency triplet by considering whether it was more or less predictable from the event n-2. For the analyses reported below, as in previous research (Howard and Howard, 1997; Song et al., 2007; Nemeth et al., 2010), two kinds of low frequency triplets were eliminated: repetitions (e.g., 222, 333) and trills (e.g., 212, 343). Repetitions and trills were low frequency for all participants and people often showed pre-existing response tendencies to them (Howard and Howard, 1997; Howard et al., 2004). By eliminating them we attempted to ensure that any high- vs. low-frequency differences are due to learning and not to pre-existing tendencies.

## Results

### Story Recall Test

**Figure 1:**
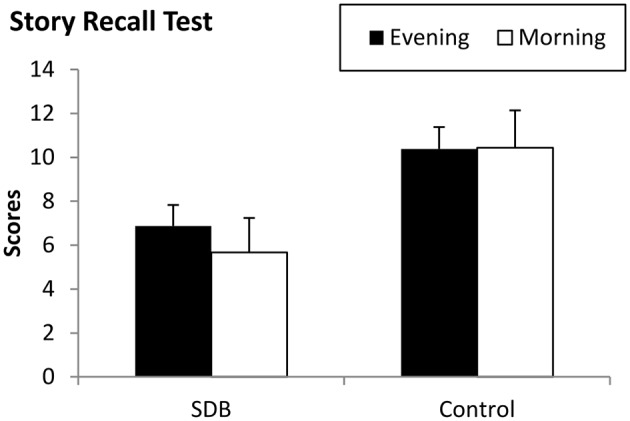
**Declarative memory performance in the evening and in the morning in the SDB and control groups.** The dependent variable was the number of correctly recalled sentences. The overall declarative memory performance of the SDB group was significantly lower compared to the control group, but there were no offline changes in the memory performance in either group. Error bars indicate SEM.

We conducted a mixed design ANOVA with SESSION (1–2) as a within-subject factor and GROUP (SDB vs. control) as a between-subject factor to assess offline changes in declarative memory performance. The main effect of GROUP was significant (*F*_(1,29)_ = 6.155, $\eta_{\text{p}}^{2}$ = 0.175, *p* = 0.019), indicating weaker story recall performance in the SDB compared to the controls (6.267 vs. 10.406, respectively). This weaker performance of the SDB group compared to the control group was evident both in Session 1 (6.87 vs. 10.38;* p* = 0.03) and in Session 2 (5.67 vs. 10.44; *p* = 0.01; Figure 1).

The main effect of SESSION failed to reach significance (*F*_(1,29)_ = 2.05, $\eta_{\text{p}}^{2}$ = 0.06, *p* = 0.16), suggesting no change in the performance during the offline period. Similarly, the SESSION × GROUP interaction was not significant either (*F*_(1,29)_ = 2.53, $\eta_{\text{p}}^{2}$ = 0.08, *p* = 0.12), suggesting no differences in offline changes between the SDB and control groups.

### Accuracy Analysis in the ASRT Task

#### Online Learning During Session 1 (Learning Phase)

**Figure 2:**
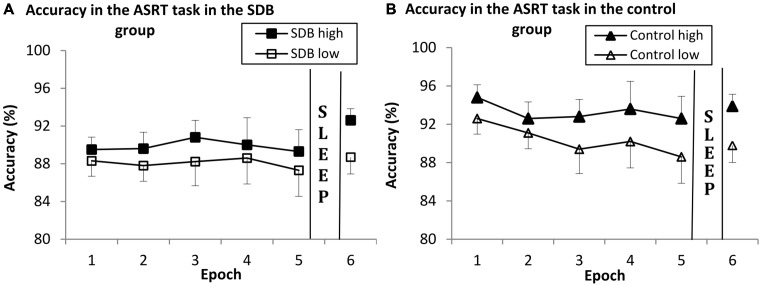
**Results of sequence-specific and general skill learning in the SDB (A) and control group (B) in Session 1 (Epoch 1–5) and Session 2 (Epoch 6) on accuracy measures.** Both groups showed significant sequence-specific and general skill learning. There were no differences in learning and in offline changes between the groups; the pattern of learning was similar in the SDB and control groups. Error bars indicate SEM.

A mixed design ANOVA was conducted on the 5 epochs of the data shown in Figure 2 with TRIPLET (2: high vs. low) and EPOCH (1–5) as within-subjects factors and GROUP (SDB vs. control) as a between-subjects factor.

There was significant sequence-specific learning (indicated by the significant main effect of TRIPLET: *F*_(1,30)_ = 61.26, $\eta_{\text{p}}^{2}$ = 0.67, *p* < 0.001), such that accuracy was greater on high- than on low-frequency triplets. SDB and control groups showed no differences in sequence-specific learning (TRIPLET × GROUP interaction: *F*_(1,30)_ = 0.29, $\eta_{\text{p}}^{2}$ = 0.01, *p* = 0.59).

The main effect of EPOCH did not reach significance (*F*_(4,120)_ = 2.58, $\eta_{\text{p}}^{2}$ = 0.07, *p* = 0.06), although accuracy decreased across epochs on a trend level. SDB and control groups performed at the same level (EPOCH × GROUP interaction: *F*_(4,120)_ = 1.29, $\eta_{\text{p}}^{2}$ = 0.04, *p* = 0.28).

The TRIPLET × EPOCH interaction was significant (*F*_(4,120)_ = 3.37, $\eta_{\text{p}}^{2}$ = 0.10, *p* = 0.01), but there were no significant differences between the groups (indicating by the TRIPLET × EPOCH × GROUP interaction *F*_(4,120)_ = 0.41, $\eta_{\text{p}}^{2}$ = 0.01, *p* = 0.79; respectively), demonstrating that the pattern of learning was similar in the groups. The main effect of GROUP did not reach significance (*F*_(1,30)_ = 3.91, $\eta_{\text{p}}^{2}$ = 0.11, *p* = 0.06), although the SDB group had lower accuracy on a trend level (SDB group: 88.6%, control group: 91.8%).

#### Offline Changes of Sequence-Specific and General Motor Skill Learning

To investigate the offline changes of sequence-specific and general motor skill learning we compared the accuracy from the last epoch of Session 1 (Epoch 5) and the epoch of Session 2 (Epoch 6) in both groups. These variables were submitted to a mixed design ANOVA with TRIPLET (2: high- vs. low-frequency) and EPOCH (2: last epoch of Session 1 and epoch of Session 2) as within-subject factors, and GROUP (SDB vs. control) as a between-subject factor. The data is shown in Figure 2.

There was significant sequence-specific learning (indicating by the main effect of TRIPLET; *F*_(1,30)_ = 95.40, $\eta_{\text{p}}^{2}$ = 0.76, *p* < 0.001), such that accuracy was greater on high- than on low-frequency triplets. It was similar in the SDB and control groups (indicated by the non-significant TRIPLET × GROUP interaction: *F*_(1,30)_ = 0.04, $\eta_{\text{p}}^{2}$ = 0.002, *p* = 0.82).

There was a significant offline changes of general motor skills (indicating by the main effect of EPOCH; *F*_(1,30)_ = 13.40, $\eta_{\text{p}}^{2}$ = 0.30, *p* = 0.01), thus accuracy increased from evening to morning. SDB and control groups performed at the same level (EPOCH × GROUP interaction: *F*_(1,30)_ = 3.26, $\eta_{\text{p}}^{2}$ = 0.09, *p* = 0.08).

The TRIPLET × EPOCH and TRIPLET × EPOCH × GROUP interactions were not significant (*F*_(1,30)_ = 0.20, $\eta_{\text{p}}^{2}$ = 0.01, *p* = 0.65; *F*_(1,30)_ = 0.28, $\eta_{\text{p}}^{2}$ *=* 0.01, *p* = 0.59; respectively), indicating that the pattern of sequence-specific learning was similar in the groups. The main effect of GROUP was not significant (*F*_(1,30)_ = 1.31, $\eta_{\text{p}}^{2}$ = 0.04, *p* = 0.26), reflecting that all groups responded with similar accuracy rates (SDB group: 88.8%, control group: 91.2%).

### Reaction Time Analysis in the ASRT Task

#### Online Learning During Session 1 (Learning Phase)

**Figure 3:**
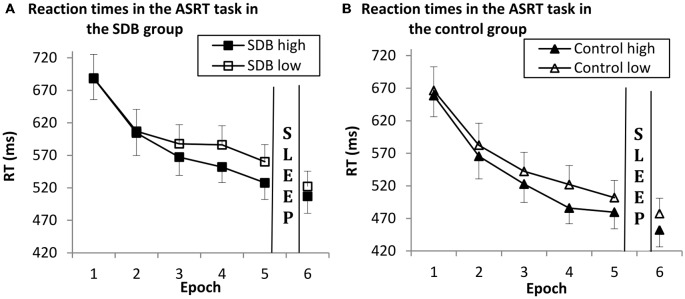
**Results of sequence-specific and general skill learning in the SDB (A) and control (B) group in Session 1 (Epoch 1–5) and Session 2 (Epoch 6) on reaction time measures.** Both groups showed significant sequence-specific and general skill learning. There were no differences in learning and in offline changes between the groups; the pattern of learning was similar in the SDB and control groups. Error bars indicate SEM.

To investigate learning during Session 1, a mixed design ANOVA was conducted on the first 5 epochs of the data shown in Figure 3, with TRIPLET (2: high- vs. low-frequency) and EPOCH (5: 1–5) as within-subject factors, and GROUP (SDB vs. control) as a between-subject factor.

Our data revealed significant sequence-specific learning (indicated by the significant main effect of TRIPLET: *F*_(1,30)_ = 64.33, $\eta_{\text{p}}^{2}$ = 0.68, *p* < 0.001), such that RTs were faster on high- than on low-frequency triplets. SDB and control groups showed no differences in sequence-specific learning (TRIPLET × GROUP interaction: *F*_(1,30)_ = 0.59, $\eta_{\text{p}}^{2}$ = 0.04, *p* = 0.44).

There was also significant general motor skill learning (shown by the significant main effect of EPOCH: *F*_(4,120)_ = 54.80, $\eta_{\text{p}}^{2}$ = 0.64, *p* < 0.001), such that RTs deceased across epochs. SDB and control groups performed at the same level (EPOCH × GROUP interaction: *F*_(4,120)_ = 0.95, $\eta_{\text{p}}^{2}$ = 0.03, *p* = 0.38).

The TRIPLET × EPOCH interaction was significant (*F*_(4,120)_ = 5.26, $\eta_{\text{p}}^{2}$ = 0.14, *p* = 0.003), suggesting that sequence-specific knowledge increased during practice. The TRIPLET × EPOCH × GROUP interaction was not significant *F*_(4,120)_ = 0.49, $\eta_{\text{p}}^{2}$ = 0.013, *p* = 0.67), indicating that the pattern of learning was similar in the groups. In overall RT both group performed at the same level (main effect of GROUP: *F*_(1,30)_ = 1.37, $\eta_{\text{p}}^{2}$ = 0.04, *p* = 0.25).

#### Offline Changes of Sequence-Specific and General Motor Skill Learning

To investigate the offline changes of sequence-specific and general motor skill learning we compared the RTs from the last epoch of Session 1 (Epoch 5) and the epoch of Session 2 (Epoch 6) in both groups. These variables were submitted to a mixed design ANOVA with TRIPLET (2: high- vs. low-frequency) and EPOCH (2: last epoch of Session 1 and epoch of Session 2) as within-subject factors, and GROUP (SDB vs. control) as a between-subject factor. The data is shown on Figure 3.

There was significant sequence-specific learning (indicating by the main effect of TRIPLET; *F*_(1,30)_ = 125.76, $\eta_{\text{p}}^{2}$ = 0.80, *p* < 0.001), thus RTs were faster on high- than low-frequency triplets when analysing the two epochs together. The groups did not differ in overall sequence-specific learning (indicated by the non-significant TRIPLET × GROUP interaction: *F*_(1,30)_ = 0.42, $\eta_{\text{p}}^{2}$ = 0.01, *p* = 0.51).

There was significant general motor skill learning during the offline period (demonstrated by the main effect of EPOCH: *F*_(1,30)_ = 20.71, $\eta_{\text{p}}^{2}$ = 0.40, *p* < 0.001), such that RTs were faster in the morning compared to the evening. The SDB and control groups showed similar level of offline general motor skill learning (EPOCH × GROUP interaction: *F*_(1,30)_ = 0.24, $\eta_{\text{p}}^{2}$ = 0.01, *p* = 0.62).

The TRIPLET × EPOCH and the TRIPLET × EPOCH × GROUP interactions were not significant* (F*_(1,30)_ = 0.84, $\eta_{\text{p}}^{2}$ = 0.02, *p* = 0.36; *F*_(1,30)_ = 2.18, $\eta_{\text{p}}^{2}$ = 0.06, *p* = 0.15, respectively), indicating that the SDB and the control group demonstrated no differences in the pattern of offline changes. There were no significant differences in the overall RTs between the SDB and control groups (main effect of GROUP: *F*_(1,30)_ = 2.54, $\eta_{\text{p}}^{2}$ = 0.07, *p* = 0.12).

## Discussion

Our goal was to investigate the consolidation of declarative and non-declarative memory in children with SDB. We believe our study to be the first to investigate the offline changes of these two types of memory processes in children with sleep disorder. We found no group difference in the consolidation of declarative memory; the SDB group, however, showed generally weaker memory performance in both sessions. We used the ASRT task to measure non-declarative learning processes. This sequence learning task allowed us to differentiate between two components of learning: general motor skill learning and sequence-specific learning. We found that these two types of non-declarative learning and consolidation are intact in children with SDB.

Our results on online declarative memory performance are in line with previous studies that found weaker declarative performance in the SDB group in general (Blunden et al., 2000; Kaemingk et al., 2003; Gottlieb et al., 2004; Kennedy et al., 2004; Csábi et al., 2013). Gottlieb et al. (2004) found lower performance on verbal and visual memory tasks in children with SDB compared to healthy controls. The mechanism causing these neuropsychological deficits has not been fully explored. Results from previous studies suggest that sleep fragmentation and intermittent hypoxia could have negative influence on the development of the central nervous system resulting structural changes in brain circuits, particularly in the hippocampus and frontal lobe (Macey et al., 2002; Bartlett et al., 2004; Halbower et al., 2006; Owens, 2009). For example Bartlett et al. (2004) found that in the left hippocampal area, N-acetyl-containing/creatine-containing compounds was significantly increased in adult OSA patients using proton magnetic resonance spectroscopic imaging. In childhood OSA Halbower et al. (2006) showed also significant differences in the mean metabolite ratio N-acetyl in the left hippocampus and right frontal cortex compared to controls leading the conclusion that childhood OSA is associated with neuronal injury in the hippocampus and frontal cortex. It is important to note that we assessed only the breathing indices during sleep. Further investigations using polysomnography need to clarify the relationship between declarative memory functions and sleep stages or sleep deprivation in children with SDB.

In the case of the overnight consolidation of declarative memory, we failed to find differences between the SDB and control group. Although there was a general group difference in the overall performance, both groups showed intact consolidation. This result contradicts with the finding of Kheirandish-Gozal et al. (2010) who demonstrated decreased consolidation of declarative memory in children with OSA. The difference between the two studies might be explained by the type of materials to be remembered (verbal vs. nonverbal) and other task characteristics (e.g., number of repetitions). Another possible explanation might be that the SDB group in our study demonstrated a floor effect with no room to forget in the offline period. For example, compared to the healthy controls, sleep disturbances in the SDB group can lead to a greater fatigue effect, which can be more pronounced by the evening where the first session took place, and could lead to weaker memory performance in the SDB group. This explanation can be tested by controlling for circadian effects and comparing AM-PM vs. PM-AM designs. Future studies need to unravel how task characteristics and/or circadian factors affect sleep-related declarative memory consolidation in children.

In the case of non-declarative learning, we found similar performance between the SDB and control group in general motor skill and sequence-specific learning in the Learning Phase, both in accuracy and in RT. Our results are in line with our previous study in which the SDB group showed impaired declarative memory performance while the non-declarative learning remained intact compared to the healthy controls (Csábi et al., 2013). Nemeth et al. (2012) using the ASRT task also found intact non-declarative sequence learning in elderly adults with OSA. These results indicate that the relationship between online non-declarative memory formation and sleep is similar in children and adults with SDB. The performance difference between declarative and non-declarative tasks in session one can be explained by that the disrupted sleep pattern influences the more attention-demanding and cortical structure-guided explicit processes (story recall), while the less attention-demanding implicit processes (ASRT task) mediated by subcortical structures are preserved (Csábi et al., 2013).

In the overnight consolidation of non-declarative memory we found no differences in the offline changes of either general motor skill or sequence-specific learning between the two groups. We found offline improvement on general motor skill, while the sequence-specific learning remained on the same level and did not improved. To our knowledge, consolidation of non-declarative memory has not been tested in children with SDB yet. These results are in line with studies investigating the effect of sleep deprivation on non-declarative sequence learning in adults without sleep disorder (Genzel et al., 2009; Van Der Werf et al., 2011). There are a few studies investigating non-declarative memory consolidation in adults with OSA. For example, Kloepfer et al. (2009) found reduced overnight improvement on average RT performance in OSA patients using a very different task compared to ours (motor adaptation vs. sequence learning, respectively). Djonlagic et al. (2012) also examined adult OSA population and revealed that OSA and control groups showed almost identical performance in the initial training in the evening on a sequence learning task, but the control group exhibited significantly more overnight improvement. The authors suggest that this weaker offline performance was caused by sleep fragmentation in OSA. In our previous study with adult OSA patients, we revealed differences in the offline changes of general motor skill learning between the OSA and control group. The control group showed offline improvement on general motor skill learning from evening to morning, while the OSA group did not. In contrast, we did not find differences between the groups in offline changes in sequence-specific learning (Csabi et al., 2014). These results partly differ from our current findings and highlight the importance of developmental factors in the consolidation of non-declarative memory: sleep disordered breathing might affect the underlying neural network differently in childhood compared to adulthood.

It worth mentioning that our study have two important potential limitations. Firstly, the declarative and non-declarative tasks could be interfere to each other. For example Brown and Robertson (2007) found that declarative tasks can actually boost non-declarative learning. It is possible that our manipulation namely counterbalancing these two types of task is not enough to eliminate the interference. Secondly, it is possible that the actual story recall task is not sensitive enough to demonstrate sleep effect. Further studies need to clarify these issues by examining the declarative and non-declarative tasks separately in different experiments and using other type of declarative tasks as well.

In conclusion, our study found dissociation between the declarative and non-declarative processes in children with SDB. Similarly with Csábi et al. (2013) we found weaker declarative memory than non-declarative performance in the first Session (Learning Phase). Regarding the consolidation, we found intact consolidation in the case of declarative memory as well as sequence-specific and general motor skill aspects of non-declarative memory in SDB. These findings imply that actual and/or long-term disturbance of sleep has a differential effect on different memory processes (online vs. offline). Our findings underscore the importance of examining the effect of sleep disturbances on motor and cognitive functions in childhood. These studies can give us a deeper insight into the effect of sleep on the developing brain and memory functions and how the relationship between sleep and memory changes from childhood to adulthood. Since persistent sleep problems in childhood can lead not only to impaired cognitive functioning—consequently lower general intelligence and school performance—but also anxiety and depression disorders in adulthood (Gregory et al., 2005), these results can help us develop more sophisticated diagnostic tools, neuropsychological profile and more effective rehabilitation programs.

## Conflict of Interest Statement

The authors declare that the research was conducted in the absence of any commercial or financial relationships that could be construed as a potential conflict of interest.
